# Treatment with FGFR2-IIIc monoclonal antibody suppresses weight gain and adiposity in KKA^y^ mice

**DOI:** 10.1038/nutd.2016.41

**Published:** 2016-11-28

**Authors:** K Nonogaki, T Kaji, T Yamazaki, Mari Murakami

**Affiliations:** 1 Department of Diabetes Technology, Tohoku University Graduate School of Biomedical Engineering, Sendai, Miyagi, Japan

## Abstract

Expression of β-Kotho, fibroblast growth factor receptor (FGFR)-1c and 2c, which bind FGF21, is decreased in the white adipose tissue of obese mice. The aim of the present study was to determine the role of FGFR2c in the development of obesity and diabetes in KKA^y^ mice. Treatment with mouse monoclonal FGFR2-IIIc antibody (0.5 mg kg^−1^) significantly suppressed body weight gain and epididymal white adipose tissue weight in individually housed KKA^y^ mice while having no effect on daily food intake. In addition, treatment with FGFR2-IIIc antibody significantly increased plasma-free fatty acid levels while having no effect on blood glucose or plasma FGF21 levels. Moreover, treatment with FGFR2-IIIc antibody had no significant effect on the expression of uncoupling protein-1, uncoupling protein-2 or peroxisome proliferator-activated receptor-γ coactivator 1α in the epididymal white adipose tissue. The treatment with FGFR2-IIIc antibody had no significant effects on daily food intake and body weight gain in individually housed KK mice. These findings suggest that FGFR2-IIIc upregulates the adiposity induced by social isolation in KKA^y^ mice, and that decreased expression and/or function of FGFR2c might be a compensatory response to enhanced adiposity. Inhibition of FGFR2-IIIc function might be a novel therapeutic approach for obesity.

## Introduction

Fibroblast growth factor 21 (FGF21) is an atypical member of the FGF family that functions as an endocrine hormone to regulate glucose and lipid metabolism.^1, 2^ Although FGF21 is reportedly produced in multiple tissues including liver, skeletal muscle, brown and white adipose tissue,^3^ circulating FGF21 is mainly derived from the liver and produced during fasting and feeding.^4, 5^ FGF21 administration increases energy expenditure, insulin sensitivity and weight loss, and normalizes glucose and lipid levels in obese and insulin-resistant rodents.^1, 6, 7, 8^

Circulating FGF21 levels, however, are elevated in obese rodents^9, 10^ and humans^11^ and the expression of β-Kotho, FGF receptor (FGFR)-1c and 2c in white adipose tissue (WAT) is decreased in obese mice.^10^ Adipose-specific FGFR1 knockout mice exhibit a normal body weight and physiological functions, but not the FGF21 treatment-induced decrease in body weight, plasma glucose, insulin and triglyceride observed in wild-type mice.^12, 13^ On the other hand, adipose-specific FGFR2 knockout mice display hypertrophic adipocytes in the mesenteric WAT but not in the subcutaneous WAT.^14^ The role of FGFR2c in the development of obesity and type 2 diabetes, however, remains unclear.

A^y^ mice with ectopic overexpression of agouti peptide, an endogenous melanocortin-4 receptor antagonist, are known to become obese and insulin-independent diabetes, when bred with KK mice.^15^ Social isolation promotes obesity due to the primary decreased energy expenditure and the insulin-independent diabetes associated with increased expression of hepatic gluconeogenic genes in KKA^y^ mice.^15^

To determine the role of FGFR2c in the development of obesity and type 2 diabetes induced by social isolation, we examined the effect of mouse FGFR2-IIIc monoclonal antibody on food intake, body weight changes, epididymal WAT (eWAT), blood glucose, and plasma-free fatty acids and FGF21 levels, and the expression of uncoupling protein-1 (UCP-1), UCP-2 and peroxisome proliferator-activated receptor-γ coactivator 1α (PGC1α) in the eWAT of individually housed KKA^y^ mice.

## Materials and Methods

Four-week-old male KKA^y^ and KK mice were purchased from Japan CLEA. Before the experiment, they were all housed (three mice per cage) with free access to water and chow pellets in a light- and temperature-controlled environment (12 h on/12 h off, lights on at 08:00; 20–22 °C). One week later, animals were transferred to individually housed conditions as described previously.^15^

Five-week-old male KKA^y^ and KK mice were intraperitoneally injected mouse monoclonal FGFR2- IIIc antibody (0.05 mg kg^−1^) or saline once per day over 6 days. The animals were provided chow pellets after the injection. Every 24 h later, body weight and food intake were measured. At the end of the 6 days, the animals were decapitated and blood was obtained for the measurement of blood glucose and plasma FFA and FGF21 levels. The eWAT was removed for the measurement of mRNA.

Mouse FGFR2-IIIc antibody was purchased from R&D Systems, Tokyo, Japan. The drugs were dissolved in 0.2 ml 0.9% saline. The dose of mouse FGFR2-IIIc used was described previously.^16^ The experiment was performed between 13:00–16:00. Whole blood was mixed with EDTA-2Na (2 mg ml^−1^) and aprotinin (500 kIU ml^−1^) to determine the plasma levels of FGF21. Blood glucose levels were measured using glucose strips (Blood glucose monitoring system; FreeStyle, KISSEI, Tokyo, Japan). The plasma FFA levels were measured by ACS-ACOD- POD assay (NEFA-SS EIKEN (Eiken Chemical co, Ltd, Tokyo, Japan). The plasma levels of FGF21 were measured by an enzyme-linked immunosorbent assay (ELISA) (rat/mouse FGF21 ELISA kits; R&D system).

The animal studies were conducted in accordance with the institutional guidelines for animal experiments at the Tohoku University Graduate School of Medicine.

### Real-Time Quantitative RT-PCR

Total RNA was isolated from mouse eWAT using the RNeasy Plus Universal Midi kit (Qiagen, Hilden, Germany) according to the manufacturer's directions. cDNA synthesis was performed using a Super Script III First-Strand Synthesis System for RT-PCR Kit (Invitrogen, Rockville, MD) using 1 ðg total RNA. cDNA synthesized from total RNA was evaluated in a real-time PCR quantitative system (LightCycler Nano Instrument Roche Diagnostics, Mannheim, Germany). The primers were used as follows: mouse UCP-1, sense, 5′-CCCAACGGCCAGTGGCCAGTCAGCG-3′, and antisense, 5′- CATGATGACGTTCCAGGACC-3′ mouse UCP-2, sense, 5′-GTTCCTCTGTCTCGTCTTGC-3′, and antisense 5′- GGCCTTGAAACCAACCA-3′ mouse PGC1α, sense 5′- GTAGCGACCAATCGGAAATC-3′ and antisense, 5′-CTAGCAAGTTTGCCTCATTCTC-3′ for mouse β-actin, sense, 5′-TTG TAA CCA ACT GGG ACG ATA TGG-3′, and antisense, 5′-GAT CTT GAT CTT CAT GGT GCT AGG-3′. The relative amount of mRNA was calculated using β-actin mRNA as the invariant control. The data are shown as the fold change of the mean value of the control group, which received saline as described previously.^15^

Data are presented as mean±s.e.m. (*n*=6). Comparisons between two groups were performed using Student's *t*-test. A *P*-value of <0.05 was considered statistically significant. Comparisons between more than two groups were performed using analysis of variance with Bonferroni's correction for multiple comparisons.

## Results

**Figure 1:**
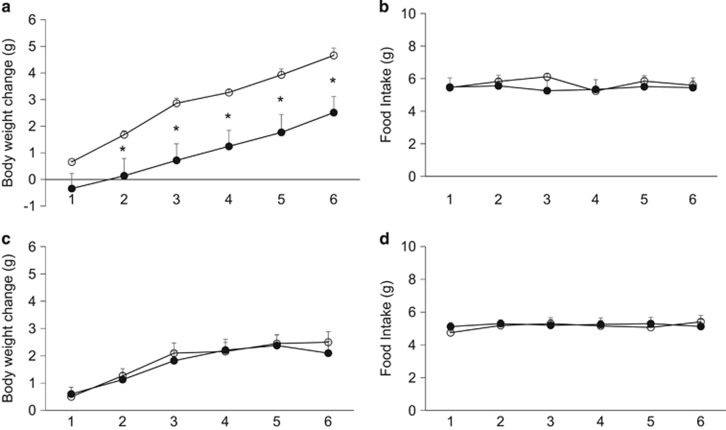
Effects of intraperitoneal injection of FGFR2-IIIc antibody (0.5 mg kg^−1^) or saline on body weight change and daily food intake in KKA^y^ mice (**a** and **b**) and KK mice (**c** and **d**). Open circles, saline control; filled circles, group treated with FGF2-IIIc monoclonal antibody; FGF2-IIIc mAb, FGF2-IIIc monoclonal antibody. Basal body weight in the saline control group and the FGFR2-IIIc antibody-treated group was 23.1±0.5 g and 23.1±0.5 g, respectively. Data are presented as the mean±s.e.m. (*n*=6/group). *P<0.05 compared with the saline control group.

**Figure 2:**
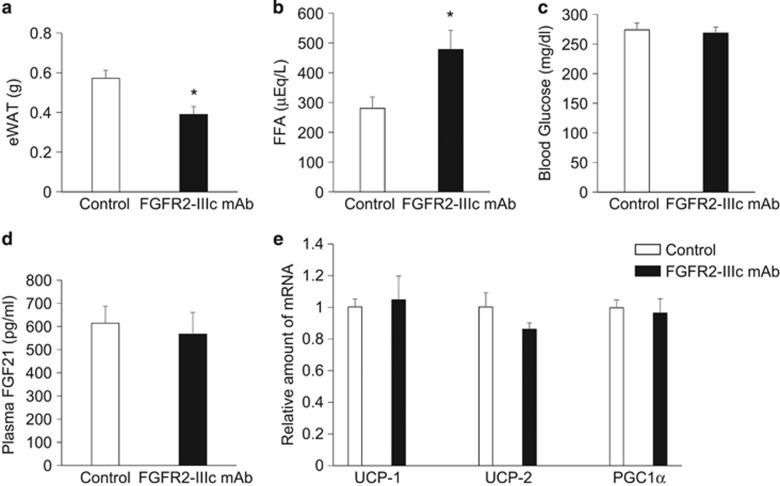
Effects of intraperitoneal injection of FGFR2-IIIc antibody (0.5 mg kg^−1^) or saline on eWAT weight (**a**), plasma FFA levels (**b**), blood glucose levels (**c**), plasma FGF21 levels (**d**) and the expression of UCP-1, UCP-2, and PGC1α in eWAT (**e**) in individually housed KKA^y^ mice. Open bars, saline control; filled bars, group treated with FGF2-IIIc monoclonal antibody; FGF2-IIIc mAb, FGF2-IIIc monoclonal antibody. Data are presented as the mean±s.e.m. (*n*=6/group). *P<0.05 compared with the saline control group.

Intraperitoneal administration of FGFR2-IIIc antibody (0.5 mg kg^−1^) significantly suppressed body weight gain in individually housed KKA^y^ mice compared with the saline control group (Figure 1a), while having no effect on daily food intake (Figure 1b). Intraperitoneal administration of FGFR2-IIIc antibody (0.5 mg kg^−1^) had no significant effects on body weight gain and daily food intake in individually housed KK mice (Figure 1c and d). In addition, treatment with FGFR2-IIIc antibody for 6 days significantly decreased eWAT (Figure 2a) and increased plasma FFA levels (Figure 2b) in individually housed KKA^y^ mice compared with the saline controls, while having no significant effect on blood glucose (Figure 2c) or plasma FGF21 levels (Figure 2d). Moreover, treatment with FGFR2-IIIc antibody for 6 days did not significantly affect the expression of UCP-1, UCP-2 or PGC1α in the eWAT (Figure 2e). Treatment with FGFR2-IIIc antibody for 6 days had no significant effects on eWAT, plasma FFA levels and blood glucose levels in KK mice (data not shown).

## Discussion

The present study demonstrated that systemic administration of FGFR2-IIIc antibody suppressed weight gain and adiposity by increased energy expenditure without suppressing food intake in individually housed KKA^y^ mice but not KK mice. In addition, these findings demonstrated that treatment with FGFR2-IIIc antibody in KKA^y^ mice increased lipolysis while having no significant effect on the expression of PGC1α, UCP-1 or UCP-2, which are involved in inducing beige fat and energy expenditure.^17, 18^

Although adipocyte-specific FGFR2c knockout mice exhibit no significant body weight alterations under a normal diet, mesenteric WAT weight is decreased.^14^ In addition, plasma FFA levels are decreased and UCP-2 expression in mesenteric WAT is increased in adipocyte-specific FGFR2c knockout mice.^14^ The adipocyte-specific FGFR2c knockout mice might have chronically increased lipolysis, leading to decreased storage of triacylglycerol in WAT.

Findings from studies in mice with FGFR1c gene knockout and monoclonal antibody-induced activation of FGFR1c suggest that FGFR1c is essential for the effects of FGF21 on body weight and glucose metabolism.^12, 13^ Our findings suggest that decreased expression and/or function of FGFR2c might not cause the FGF21-resistant state, but might rather be a compensatory response to enhanced adiposity.

Despite reduced adiposity, treatment with FGFR2-IIIc antibody did not suppress hyperglycemia induced by social isolation in KKA^y^ mice. Because individually housed KKA^y^ mice display increased hepatic gluconeogenesis,^15^ treatment with FGFR2-IIIc antibody might not affect the increased hepatic gluconeogenesis. It remains unclear whether FGFR2-IIIc antibody has a direct action on WAT or the central nervous system (CNS)-mediated action. Because there are very low levels of FGFR2c mRNA in the hypothalamus compared with FGFR1c and FGFR3c,^19^ the suppressive effect of FGFR2c antibody on body weight gain and adiposity might be due to the direct action on WAT rather than the CNS-mediated action.

In summary, these findings suggest that the treatment with FGFR2-IIIc antibody suppresses body weight gain and adiposity without affecting food intake and hyperglycemia in individually housed KKA^y^ mice. Treatment with FGFR2-IIIc antibody increased lipolysis while having no effect on the expression of genes involved in inducing beige fat and energy expenditure. Inhibition of FGFR2-IIIc function may be a novel therapeutic approach for obesity.
